# *MIR133A* in Cancer Biology: Target Genes, Biological Effects, and Biomarker Potential

**DOI:** 10.3390/genes17070781

**Published:** 2026-07-05

**Authors:** Grinsun Sharma, Santosh Lamichhane, Soo-Cheon Chae

**Affiliations:** 1 School of Biomedical Sciences, Kent State University, Kent, OH 44240, USA; grinsun58@gmail.com; 2 Department of Genome Sciences, University of Virginia, Charlottesville, VA 22903, USA; lakki38786@gmail.com; 3 Department of Pathology, School of Medicine, Wonkwang University, Iksan 54538, Republic of Korea

**Keywords:** *MIR133A*, biomarker, apoptosis, cell migration

## Abstract

Cancer is one of the leading causes of mortality and morbidity worldwide. Various studies have highlighted the involvement of microRNAs (miRNAs) in tumor initiation and progression. MiRNAs are endogenous, non-coding, single-stranded RNA molecules that interact with the 3′-untranslated region (3′-UTR) of target mRNAs to inhibit mRNA translation or promote mRNA degradation. Various studies have reported that *MIR133A* is expressed at reduced levels in many tumor tissues and inhibits tumor progression. In this review, we comprehensively summarize the interactions of *MIR133A* and its target genes in the most commonly diagnosed cancers, namely, breast, lung, colorectal, gastric, and prostate. These results demonstrated that *MIR133A* is one of the optimal biomarkers for the diagnosis, prognosis, and prediction of various tumors, providing insights into the clinical management and practice of malignant tumors.

## 1. Introduction

Cancer is a major contributor to mortality and morbidity worldwide [1]. In 2020, approximately 20 million novel cases and about 10 million cancer related mortalities were recorded [2]. Breast cancer is ranked as a primary cancer type, followed by lung cancer and colorectal cancer (CRC). Lung cancer (18%) accounts for the highest proportion of cancer-related deaths, followed by CRC (9.4%) [3]. Cancer incidence is not only affected by family history, environmental factors, and genetic susceptibility; it also increases with age, and it is affected by obesity and unhealthy diets.

MiRNAs are endogenous, noncoding single-stranded RNA molecules that interact with the 3′-untranslated region (3′-UTR) of their target gene, leading to either inhibition of mRNA translation or acceleration of mRNA degradation [4]. MiRNAs influence cancer pathology by behaving either as oncogenes or tumor suppressor genes [5]. Normally, miRNAs act through feedback mechanisms to safeguard cell growth, differentiation, and apoptosis. However, dysregulation of miRNA expression can significantly alter the expression of other genes, thereby promoting the transformation of normal cells into cancerous cells [6]. Roughly half of human genes are controlled by internal miRNAs, and their abnormal expression leads to diverse biological changes, including apoptosis, cell differentiation, proliferation, migration, invasion, and angiogenesis [7]. Various studies have shown that miRNA regulation plays a significant role in tumor initiation and advancement [8]. Furthermore, miRNAs have surfaced as a promising factor in early disease detection, categorization, and treatment; however, their validation and quantification are still challenging in clinical settings [9].

*MIR133* has been identified in mice, flies, and many mammalian species, including humans [10]. According to reports, *MIR133* possesses three identified human gene loci, with two gene loci (bicistronic) for *MIR133A* situated on chromosomes 18 (*MIR133A1*) and 20 (*MIR133A2*), and the third for *MIR133B* is situated on chromosome 6 [11]. The members of MIR133 have different sub-types, varying across different species. In humans, the *MIR133* family has three subtypes (*miR-133a-3p*, *miR-133a-5p*, and *miR-133b*) [12]. *MIR133A* exhibits high expression levels in both the heart and skeletal muscles, and *MIR133B* displays high expression levels in the skeletal muscle. In addition, *MIR133A* and *MIR133B* belong to the same miRNA family, but they differ in terms of genomic origin, tissue distribution, and possibly cancer-specific function. Therefore, their roles should not be considered completely interchangeable [13,14]. However, the most abundantly expressed form of *MIR133* is *miR-133a-3p* (hereby referred to as *MIR133A* in this study) in all species.

Studies on genomic expression on miRNAs have shown that *MIR133A* is prevalent across a range of tissues or organs and downregulated in cervical cancer [15], CRC [16], human glioma [17], non-small cell lung cancer [18], breast cancer [19], ovarian cancer [20], retinoblastoma [21], gastric cancer (GC) [22], and prostate cancer (PC) [23].

*MIR133A* is upregulated in human airway epithelial cells, contributing to epithelial mesenchymal transition (EMT) [24], and its expression, along with *miR-1*, is also increased in multiple myelomas compared with normal samples [25].

## 2. MicroRNA Biogenesis, Structure, and Biology

**Figure 1:**
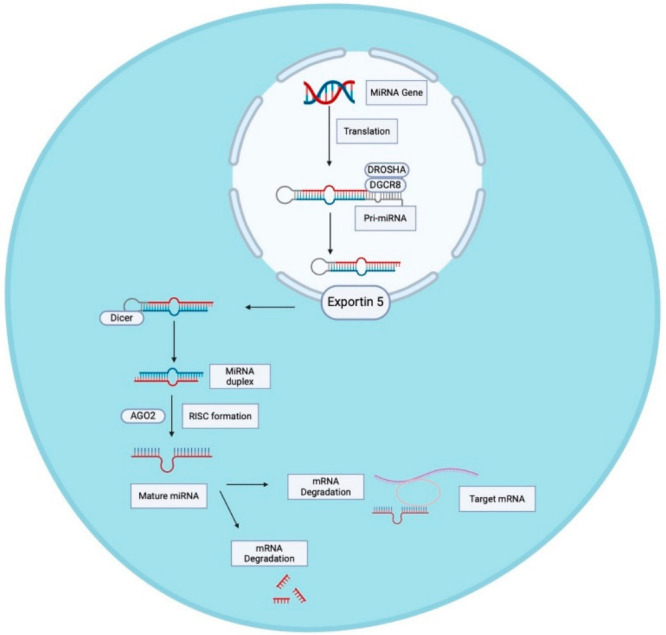
Schematic diagram of the mechanism of biogenesis of microRNA. The biogenesis started at the nucleus, where the miRNA gene is translated to a double-stranded loop by RNA polymerase, which is cleaved by Drosha-DGCR8 complex to form a shorter miRNA. Exportin-5 transports the shorter miRNA to the cytoplasm, where it is processed using Dicer. After the formation of RISC complex, that unwinds mature miRNA, mature miRNA forms that recognizes and binds to the target complementary sequence of 3′ UTR of mRNAs. Image was created in BioRender Lamichhane, S. (2025).

In summary, the biosynthesis of miRNA starts at the nucleus, as the single-stranded miRNA, which requires high sequence complementarity, is converted using RNA polymerase II/III to a double-stranded loop like pri-miRNA (≃100–120 nucleotides) (Figure 1). Also, the microprocessor complex (Drosha and DGCR8) recognizes and cleaves the pri-miRNA to create shorter precursor miRNA (pre-miRNA ≃70 nucleotides). Exportin-5 transports the pre-miRNA to the cytoplasm, where it undergoes processing using Dicer to produce mature miRNA.

This matured miRNA duplex is inserted into the argonaute2 (AGO2) family proteins, thereby forming a complex known as miRNA-induced silencing complex (miRISC). The binding of mature miRNA to the RISC complex causes the unwinding of mature miRNA with the help of a special domain. In contrast, one strand (guide strand) of the mature miRNA remains with AGO2 protein while the other (passenger strand) is broken down; for some miRNAs, the passenger strand can also be activated. Then, a single active guide complex (≃15–20 nucleotides) recognizes and binds to the target complementary sequence of the 3′ UTR of mRNAs. As a result, mRNA is cleaved, mRNA translation is repressed, and mRNA degradation is induced [26].

The most critical determinant region for miRNAs and their target mRNAs is the “seed region” that is mostly situated at positions 2–8 from the miRNA 5′-end [27]. Recent studies have shown that perfect binding between miRNAs and mRNAs at the “seed region” upholds the functional equilibrium of gene networks within cells [28]. However, mRNA sites that show incomplete binding can still act as effective targets for miRNAs, meaning that they are still highly likely to be false-positive in bioinformatics prediction [29]. Therefore, the target sites predicted using bioinformatics need to be confirmed through experiments.

## 3. *MIR133A* in Cancer

More recently, it has been reported that miRNAs govern about fifty percent of human genes and are aberrantly expressed in tumor tissues, waste products, and body fluids from cancer patients [30]. Numerous studies have shown that the regulation of *MIR133A* contributes to malignancies, including cancer progression. Recently, studies on miRNAs have illuminated their potential roles in the initiation and advancement of cancer. In recent decades, various studies have shown that miRNAs regulate multiple target genes and are associated with tumors and are differentially expressed in different cancers [15,16,17,18,19,20,21,22,23,24,25].

In this review, we provide an overview of current knowledge regarding the significance of *MIR133A* in different cancers, focusing on its functional interaction with putative target genes and the relevant pathways.

### 3.1. MIR133A in Colorectal Cancer (CRC)

CRC is a widespread gastrointestinal malignancy, known as the second-highest cause of cancer-related mortality and the third-highest cancer incidence [31]. CRC incidence is growing swiftly in Asia, which was previously known as a low-risk region [32]. The precise mechanism of CRC remains unclear, though various studies have reported that cellular metabolism, genetic modification, and mutation are vital factors for pathogenesis [33]. The progression of intestinal tumors occurs through distinct clinical phases, intricately linked to specific genetic mutations. The fate of intestinal epithelial cells is governed by the Wnt/β-catenin cascade. APC, β-catenin, and KRAS/BRAF mutations lead to adenoma growth. Further, inactivating TGF-β mutations introduce malignant features, while p53 mutations drive transformation into adenocarcinoma. Additionally, the activation of Cox-2, epidermal growth factor, and VEGF is correlated with CRC development and disease advancement [34].

**Figure 2:**
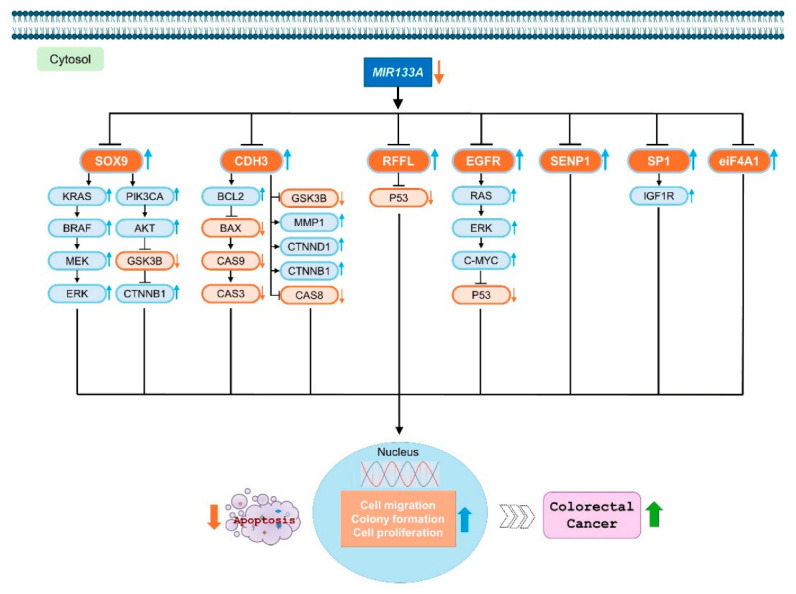
Schematic of the putative mechanism of *MIR133A* in CRC. Downregulation of *MIR133A* expression leads to an increase in the expression of its target genes SOX9, CDH3, RFFL, EGFR, SENP1, SP1, and eiF4A1. The upregulation of these genes directly or indirectly activates downstream or related pathways such as KRAS, PIK3CA, BCL2, MMP1, p53, RAS, and IGF1R, leading to decreased apoptosis and increased cell migration and metastasis.

Accumulating evidence suggests that *MIR133A* inhibits CRC by inhibiting cell proliferation and promoting apoptosis (Figure 2). *MIR133A* complexly regulates the SOX9 gene, which in turn affects the PIK3CA-AKT1-GSK3B-CTNNB1 and KRAS-BRAF-MAP2K1-MAPK1/3 pathways. This leads to the inhibition of growth, migration, and colony formation in CRC cells. Notably, PIK3CA and KRAS pathways, recognized for the role they play in cell proliferation, also function as oncogenes [35].

Tumor cells typically evade apoptosis pathways, but *MIR133A* counteracts CRC growth by promoting apoptosis and suppressing cell proliferation. This occurs through regulating RFFL gene, causing G0/G1-phase arrest, and the activation of tumor suppressor p53/p21 cascade. G0/G1-phase arrest halts the cell cycle at the G0 or G1 phase, suppressing DNA synthesis and effectively suppressing overall cell proliferation [36]. Furthermore, *MIR133A* has great significance in reducing the levels of the oncogenic RAS/ERK/MYC pathway through epidermal growth factor receptor (EGFR) modulation, leading to increased p53 expression. It also regulates EMT factors, effectively suppressing CRC proliferation, metastasis, and chemoresistance [37]. CDK (cyclin-dependent kinase) governs cell cycle progression with cyclins, and its dysregulation can lead to cancer cell proliferation. CDK inhibitors show promise in treating colon cancer by disrupting cell cycle control. Additionally, *MIR133A* inhibits SENP1 expression, upregulating CDK inhibitors like p16, p19, p21, and p27, ultimately inhibiting cell proliferation [38] and colony formation by regulating Sp1 transcription factor (SP1) and IGF1R gene [39]. Cadherins (CDH3) are crucial for cell adhesion, and it is aberrantly expressed in CRC due to promoter hypomethylation. *MIR133A* regulates CDH3-mediated catenin, matrix metalloproteinases (MMPs), apoptosis, and the EMT pathway, thereby suppressing cell growth, migration, and colony formation in CRC. Therefore, further investigation is needed to explore the potential involvement of *MIR133A* in CRC [16].

### 3.2. MIR133A in Gastric Cancer

**Figure 3:**
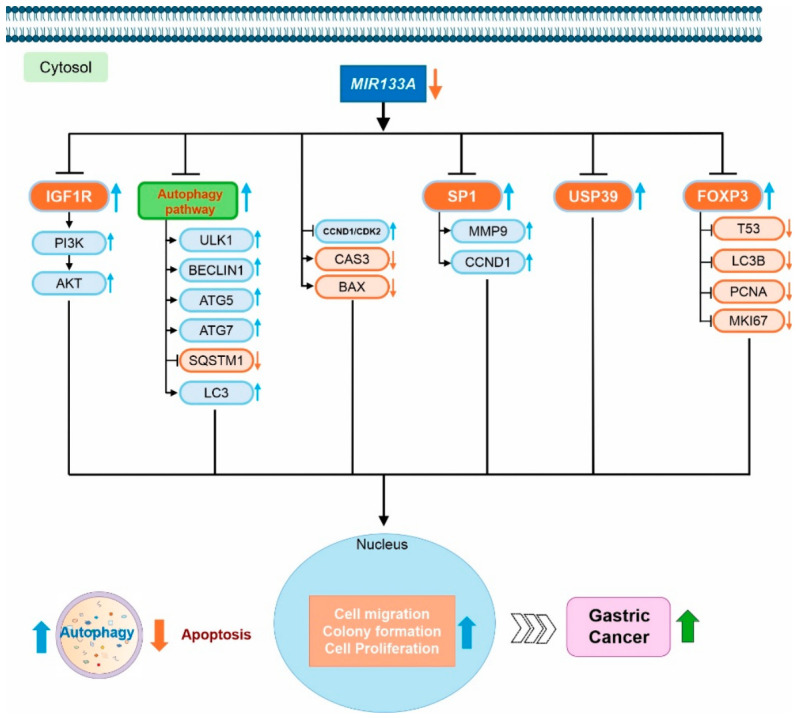
Schematic diagram of the putative mechanism of *MIR133A* in GC. Downregulation of *MIR133A* expression leads to increased expression of IGF1R, SP1, USP39, and FOXP3. Upregulations of these genes directly or indirectly activate downstream or associated pathways, such as PI3K, CAS3, MMP9, and LC3B, leading to a decrease in apoptosis and increase in metastasis of GC.

In recent times, gastric cancer (GC) has emerged as a prominent contributor to cancer-related mortality. The survival rates for individuals with GC remain relatively low, despite the development of numerous treatments and drugs [40]. Distal gastric cancer is primarily associated with H. pylori infection and dietary factors, while proximal gastric cancer is linked to gastroesophageal reflux disease (GERD) and obesity; these are the main causative agents. Research has shown a significant dose-dependent correlation between smoking and the likelihood of having GC [41]. Also, the initiation of GC is complex and multifactorial. It includes genetic, epigenetic, and environmental factors such as diets, microbes, and their metabolites. Previous studies have implicated *MIR133A* in regulating GC initiation and development (Figure 3). Hu et al. found that bufothionine, a sulfur-containing compound, upregulated *MIR133A*, which amplifies the suppression of GC by deactivating the eIGF1R/PI3K/AKT cascade and increases the apoptosis and production of reactive oxygen species, which are reversed by downregulating *MIR133A* [42]. Also, SP1, a transcriptional factor, is a direct target of *MIR133A* that binds to the 3′-UTR region. It was reported that *MIR133A* negatively regulates SP1 and its downstream molecules MMP9 and cyclin D1 (CCND1), which limits proliferation, invasion, and migration in GC [22]. The luciferase assay verified that USP39 was a direct target of *MIR133A*, and there was an inverse relation between them. A higher expression of *MIR133A* causes the downregulation of USP39, which inhibits cell proliferation and could be the novel therapeutic target for gastric cancer prognosis [43]. However, Li et al. showed that cancer cells utilize autophagy to maintain mitochondrial energy and function, maintaining requirements for growth and proliferation, ultimately promoting their survival during starvation conditions. *MIR133A* is involved in GC proliferation by regulating the expression of FOXP3, which in turn increases the expression of proliferation markers (PCNA and MKI67) and apoptosis-related marker (TP53) and autophagy marker (LC3B) [44].

### 3.3. MIR133A in Lung Cancer (LC)

LC is the most prevalent cancer and primary contributor to tumor related death. LC has a tendency to be asymptomatic for a long time, meaning that patients with major disease are often diagnosed at an advanced stage [45]. Risk factors include, smoking, genetic factors, previous respiratory disease, various viral infections, i.e., human papillomavirus, occupational exposure to carcinogens, diet and obesity, air pollution, and family history of cancer [46].

**Figure 4:**
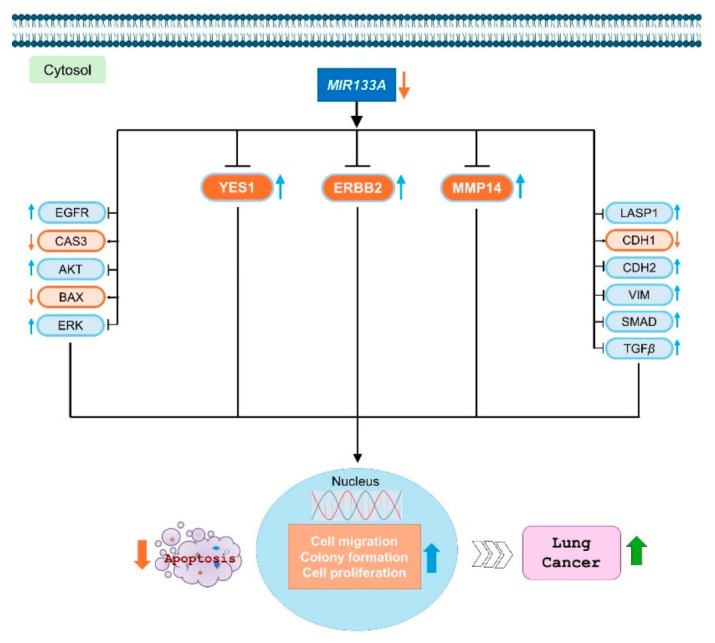
Schematic diagram of the putative mechanism of *MIR133A* in LC. Downregulation of *MIR133A* expression leads to an increase in the expression of YES1 and ERBB2. Upregulation of these genes directly or indirectly activates downstream or associated pathways, such as EGFR, CAS, CDH1, and CDH2, leading to a decrease in apoptosis and increase in metastasis of LC.

LC is a multifaceted and iterative process that induces the gradual accumulation of molecular and genetic anomalies. In the case of LC, about 2 million new cases are identified annually, resulting in 1.7 million fatalities. As the malignancies have a diverse nature, the analysis of microRNA gives us an idea of the type of cancer [47]. Evidence has shown that *MIR133A* is reduced in lung cancer patients, (Figure 4), which results in poor clinical outcomes, prognosis, and lower survival rates [48]. Meanwhile, *MIR133A* has been found to be highly upregulated in the serum samples of patients with lung adenocarcinoma (LA) compared with the controls; however, the variation was non-significant in cancer tissues [49]. In contrast to LA, *MIR133A* expression was downregulated in NSCLC and EGFR was higher than normal mucosa. Furthermore, the restoration of *MIR133A* in NSCLC cells suppressed cell growth, induced apoptosis, and suppressed the EGFR/AKT/ERK signaling pathway [50].

Shen et al. showed that, *MIR133A* also inhibits cell proliferation of NSCLC cells by regulating YES proto-oncogene 1 (YES1). The relationship between the *MIR133A* and YES1 gene was investigated using a luciferase assay. Also, the increase in YES1 expression was correlated with a poor prognosis and worse clinical outcome of NSCLC [51]. Another study showed that *MIR133A* levels were downregulated in NSCLC, while LASP1 (LIM and SH3 domain protein 1) expression, a direct target of *MIR133A*, increased. They reported that *MIR133A* overexpression inhibited cell viability, EMT, and TGF-β/Smad3 pathways and suppressed tumor growth by regulating LASP1 expression [52]. ERBB2 (receptor tyrosine-protein kinase erbB-2) is a member of the EGFR family and involved in proliferation, differentiation, and apoptosis. It was a direct target of *MIR133A*, and upregulation of *MIR133A* suppressed growth, migration, and invasion of NSCLC cells by regulating ERBB2 [53].

### 3.4. MIR133A in Breast Cancer (BC)

BC is one of the most common malignant cancers, resulting in 14% of breast cancer-related deaths in women. There is a great disparity in cancer patients’ survival rates; for example, the five-year survival rate in the developed countries is 80%, whereas in developing countries the rate of survival is below 40%. Also, in American females, the risk of developing breast cancer is about 10% [54]. Risk factors include age, timing of menarche and menopause, age at first pregnancy, family medical history, exposure to radiation, and lifestyle choices [55]. There is a limited understanding of the mechanism of breast cancer, underlying the need for novel strategies based on molecular mechanisms to be developed. Numerous studies have reported that *MIR133A* was significantly decreased in breast cancer [19,56,57].

**Figure 5:**
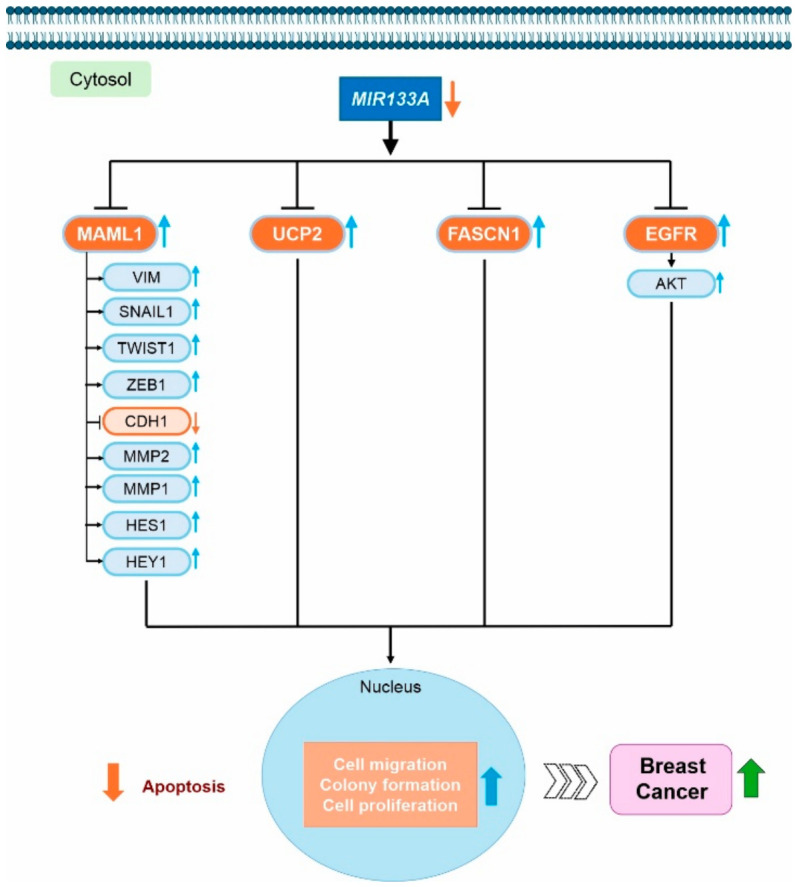
Schematic diagram of the putative mechanism of *MIR133A* in BC. Downregulation of *MIR133A* expression leads to increase in MAML1, UCP2, FASCN1, and EGFR expression. Upregulations of these genes directly or indirectly activate downstream or associated pathways, such as VIM, CDH1, AKT, and MMP2, leading to decrease in apoptosis and increase in metastasis of BC.

The accumulated research suggests that *MIR133A* acts as a tumor suppressor and enhances cell cycle arrest in the G2/S phase by targeting EGFR and its downstream AKT pathway. Thus, cell cycle and growth were inhibited via the EGFR/AKT signaling pathway (Figure 5). Likewise, Shi et al. showed that *MIR133A* was remarkably suppressed in BC cells and tissues. MAML1 (mastermind-like transcriptional coactivator 1), a notch signaling coactivator, was confirmed as a direct target of *MIR133A* and is reduced by binding to the 3′-UTR region, which inhibits invasion, EMT, and metastasis in an in vivo models [58]. Along with that, Yuan et al. have shown that *MIR133A* plays a functional role in BC cells resistant to doxorubicin by controlling the expression of UCP-2 (uncoupling protein 2) [59]. In BC, *MIR133A* was reported to downregulate, resulting in lymph node metastasis and reduced survival rates of patients, whereas, the restoration of *MIR133A* suppresses BC cell growth and invasion by targeting FASCN1 gene [57]. As such, many studies have suggested the use of *MIR133A* as a biomarker and therapeutic target. Although *MIR133A* has been reported to be downregulated in several cancer types, acting as a tumor suppressor, others have shown that circulating *MIR133A* is upregulated in BC patients [60].

### 3.5. MIR133A in Prostate Cancer (PC)

**Figure 6:**
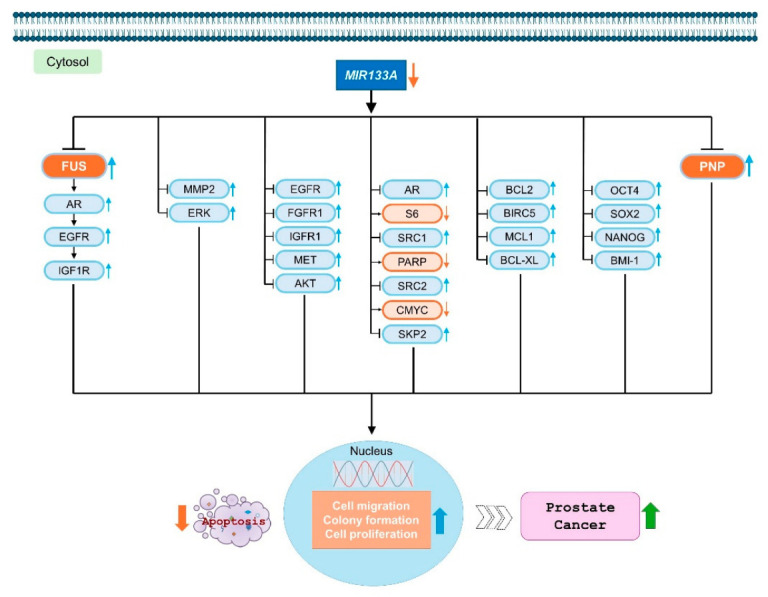
Schematic diagram of the putative mechanism of *MIR133A* in PC. Downregulation of *MIR133A* expression leads to increase in FUS, PNP, and MMP expression. Upregulation of these genes directly or indirectly activates downstream or associated pathways, such as AR, S6, ERK, EGFR, and SOX2, leading to decrease in apoptosis and increase in metastasis of PC.

PC is the second most commonly identified, and a leading cause of tumor linked death among males. Older men over 65 years of age are most vulnerable to developing PC, and it was estimated that the disease caused about 400,000 deaths in 2022 [61]. Risk factors include age, family history, obesity, physical activity, smoking, and occupational exposures to chemicals hazards [62]. As with various other cancers, studies have highlighted our limited understanding of PC, emphasizing the need for research focused on novel prevention strategies. In this context, microRNAs have demonstrated important regulatory roles in PC. Studies have reported that *MIR133A* is often deregulated in cancer, and its low expression has been shown to be associated with recurrence and metastasis in PC (Figure 6). Both the androgen receptor and FUS (fused in sarcoma), a protein that interacts with the androgen receptor, were found to be direct targets and downstream of *MIR133A*. Overexpression of *MIR133A* significantly inhibits androgen receptor-linked growth in PC cells by regulating androgen receptors and FUS, thereby modulating androgen and its downstream receptors (IGFR and EGFA) [63]. Tang et al. showed that *MIR133A* was reduced in PC, and the overexpression of *MIR133A* inhibits PC bone metastasis by regulating PI3K/AKT cascade via the regulation of several cytokine targets like EGFR, IGF1R, and MET [23]. Also, MCL1 is a target of *MIR133A*, and its overexpression is directed to the suppression of MCL1, which inhibits cell proliferation and loss of chemoresistance to docetaxel. Moreover, other mRNAs, such as FASCN1, LASP1, MMP14, IGF1R, and GSTP1, are thought to be simultaneously regulated by *MIR133A*, which may have clinical significance as biomarkers and prognostic and therapeutic agents for aggressive PC [64].

Table 1 classifies the summarized study results into in vitro evidence, in vivo evidence, and clinical patient evidence, taking into account differences in the reliability of evidence across the reviewed studies. This classification allows for a clearer interpretation of *MIR133A* dysregulation, reported target genes, and biological effects across various cancer types.

## 4. *MIR133A* as Potential Diagnostic and Prognostic Biomarker

MicroRNA emerged as a significant cancer hallmark after its identification in 2008 [65,66], with potential as a biomarker across various diseases, including cancer. The perfect biomarker ought to be simply accessible, highly specific, reliable, and reproducible, with high sensitivity often extracted through liquid biopsies such as urine, serum, blood, feces, and other bodily fluids. Despite recent advancement, the study of miRNAs is still in the inception phase. MiRNA expression varies across cancers, enabling precise classification and diagnosis [67]. MIR133A is promising as a diagnostic, prognostic, and predictive biomarker in tumor management, providing valuable insights for clinical decision-making and therapeutic implementation. Studies have shown its downregulation in various cancers, indicating its potential for early identification, which is crucial for improving five-year survival rates. For instance, Peterson et al. showed in oral squamous cell carcinoma that *MIR133A* was aberrantly expressed compared with control cell lines [68]. *MIR133A* shows higher expression in lymphoma-associated hemophagocytic syndrome (LAHS) compared with benign diseases related to hemophagocytic lymphohistiocytosis, indicating its potential as a diagnostic marker for LAHS. However, further validation through clinical studies is necessary to confirm its efficacy and reliability [69]. *MIR133A* expression also correlates with conditions beyond cancer, such as atherosclerosis, arterial stiffness, vascular smooth muscle cell differentiation, apoptosis, inflammation, cardiac fibrosis, endothelial function and angiogenesis [70], and acute myocardial infraction (AMI) [71]. In breast cancer, downregulation of *MIR133A* is significantly linked with clinical stage, metastasis, and survival time [57]. ZiaSarabi et al. showed that *MIR17*, *MIR25*, and *MIR133B* have been proposed as biomarkers for the diagnosis of GC based on their expression levels compared with controls [72]. Furthermore, *MIR133A* expression differs between liver metastases and primary tumors, suggesting its potential in grading gastrointestinal neuroendocrine neoplasms [73]. Additionally, plasma levels of *MIR133A* are linked to blood pressure monitoring and ambulatory blood pressure parameters, potentially serving as potential prognostic biomarkers for white-coat hypertension detection [74].

Moreover, miRNAs exhibit stability and easy identification in serum, facilitated by their association with AGO2, micro-vesicle envelopment, and assistance from various proteins and lipids, ensuring their circulation in the bloodstream [75]. Hence, the multifaceted roles of microRNAs, particularly *MIR133A*, in cancer diagnosis, prognosis, and beyond underscore their significance as promising biomarkers. Their differential expression patterns offer valuable insights into disease classification and management, with potential applications extending to cardiovascular and neuroendocrine conditions. Further validation through clinical studies is crucial to fully realize their diagnostic and prognostic utility.

## 5. Therapeutic Potentials

MiRNAs have significant therapeutic potential across different ailments, like tumors, cardiovascular disease, nervous system disorder, and infectious diseases. They can regulate gene expression and are targeted for therapies like miRNA replacement or inhibition to modulate disease processes. Challenges such as efficient delivery and off-target effects remain, but ongoing research aims to overcome these hurdles for clinical translation. Detecting miRNAs that are stably released into body fluids with a high level of sensitivity, specificity, and robustness in a non-invasive diagnostic method is challenging [76]. Various techniques, including real-time PCR, miRNA microarray assay, in situ hybridization, Western and Northern blots, and next generation sequencing (NGS) have been proposed to enhance miRNA detection sensitivity and specificity, yet specific target detection remains a challenge [77].

Despite these challenges, miRNAs have shown immense potential in cancer therapy. *MIR133A*, for instance, has demonstrated a significant role in inhibiting cancer both in vivo and in vitro; it exhibits multifaceted roles in inhibiting cancer progression through various mechanisms. Additionally, *MIR133A* deregulates angiogenic properties like proliferation rate, cell viability, migration [78], and adipocyte browning in vitro [79]. Furthermore, *MIR133A* obstructs the growth of in vitro triple-negative BC cells and inhibits lung adenocarcinoma metastasis and cell invasion by regulating genes like FLOT2 and YES1 [80]. Xiong et al. identified activated C kinase 1 receptor (RACK1) as a target gene of *MIR133A* and found that the transfection of glioma cells with *MIR133A* inhibited migration and invasion with increased cell death [81]. These data show that there is a strong reason to use *MIR133A* analogs in cancer treatment. While microRNA-based therapies are still in their infancy, ongoing research and accumulated experience in this field hold promise for future advances in tumor-specific microRNA-related cancer diagnosis and treatment.

## 6. Conclusions

This study shows that *MIR133A* is suppressed in various tumors and the downregulation of *MIR133A* is associated with cancer progression, growth, and metastasis. Various targets of *MIR133A* are being identified using the dual luciferase assay; however, the exact mechanisms and downstream inhibitory pathways remain unknown, suggesting the need for a combination of bioinformatics tools and wet-lab research to identify tumor-specific miRNA targets with prolonged effects. While this study focuses on the significance of *MIR133A* in the prognosis and diagnosis of malignancies, its targeted and cancer-specific use is still in the early stages. Therefore, we should be more focused on the clinical significance of *MIR133A*.

## Figures and Tables

**Table 1:** Target genes and biological effects of *MIR133A* in major cancers.

Cancer Type	*MIR133A* Expression	Target Genes/Pathways	Biological Effects	Evidence Classification
Colorectal cancer (CRC)	Downregulated	SOX9, RFFL, EGFR, SENP1, SP1, CDH3; associated with PI3K/AKT-, KRAS/MAPK-, and p53/p21- related pathways	Suppresses cell proliferation, migration, colony formation, metastasis, EMT, and chemoresistance; promotes apoptosis and G0/G1 cell-cycle arrest	Mainly in vitro functional evidence; some clinical/tissue-expression evidence
Gastric cancer (GC)	Downregulated	IGF1R, SP1, USP39, FOXP1, PI3K/AKT, MMP9, CCND1, and autophagy-related markers including LC3B	Inhibits proliferation, invasion, and migration; promotes apoptosis; may also regulate autophagy-associated survival depending on context	Mainly in vitro evidence; limited clinical/prognostic evidence
Lung cancer/NSCLC	Downregulated in NSCLC and lung cancer tissues	EGFR, YES1, LASP1, ERBB2, AKT/ERK, and TGF-β/SMAD pathways	Suppresses cell growth, viability, EMT, migration, invasion, and tumor growth; induces apoptosis; associated with prognosis and survival	In vitro, in vivo, and clinical patient evidence
Breast cancer (BC)	Mostly downregulated in tissues/cells; circulating *MIR133A* reported as upregulated in some patient samples	EGFR, MAML1, UCP-2, FASCN1, AKT, Notch/EMT, and drug-resistance pathways	Inhibits cell growth, invasion, EMT, metastasis, and doxorubicin resistance; promotes cell-cycle arrest; associated with lymph node metastasis and poor survival	In vitro, in vivo, and clinical patient evidence
Prostate cancer (PC)	Downregulated; low expression associated with recurrence and metastasis	AR, FUS, EGFR, IGFR1, MET, MCL1, FASCN1, LASP1, MMP14, GSTP1, PI3K/AKT, and androgen receptor signaling	Suppresses androgen receptor-driven proliferation, bone metastasis, cell proliferation, and docetaxel chemoresistance; potential prognostic and therapeutic relevance	Mainly in vitro and clinical/metastasis-associated evidence; some functional pathway evidence
Other reported cancers including cervical cancer, glioma, ovarian cancer, retinoblastoma, oral squamous cell carcinoma, lymphoma-associated hemophagocytic syndrome, and gastrointestinal neuroendocrine neoplasms	Mostly downregulated in several tumors; upregulated in LAHS and some circulating samples	SOX4, EGFR, CREB1, RACK1, and other tumor-specific targets	Regulates proliferation, apoptosis, migration, invasion, diagnostic classification, and prognostic potential	Mixed evidence: in vitro, clinical patient, and biomarker-based studies

## Data Availability

No new data were created or analyzed in this study.

## Abbreviations

The following abbreviations are used in this manuscript:

3′ UTR	3′ Untranslated region
AKT	AKT serine/threonine kinase
BRAF	B-Raf Proto-Oncogene, Serine/Threonine Kinase
BC	Breast cancer
CAS	Caspase
CDH1	E cadherin
CDH2	N Cadherin
CRC	Colorectal cancer
CTNNB1	Beta catenin
EGFR	Epidermal growth factor receptor
EMT	Epithelial–mesenchymal transition
ERBB2	Human epidermal growth factor receptor 2
ERK	Extracellular signal-regulated kinase
FASCN1	Fascin actin-bundling protein 1
FOXP3	Forkhead box O3
FUS	Fused in sarcoma
GC	Gastric cancer
GSK3B	Glycogen synthase kinase 3 beta
IGF1R	insulin-like growth factor 1 receptor
LAHS	Lymphoma-associated hemophagocytic syndrome
LC	Lung cancer
LC3B	Microtubule-associated protein 1 light chain 3
MAML	Mastermind-like 1
MAPK	Mitogen-Activated Protein Kinase
MiRISC	miRNA-induced silencing complex
MMP	Matrix metalloproteinases
PC	Prostate cancer
*MIR133A*	MicroRNA 133A
PI3K	Phosphatidylinositol 3-kinase
SOX2	SRY-box transcription factor 2
VEGF	Vascular endothelial growth factor
VIM	Vimentin
YES1	YES proto-oncogene 1
